# Etiology and Management of Pediatric Intestinal Failure: Focus on the Non-Digestive Causes

**DOI:** 10.3390/nu13030786

**Published:** 2021-02-27

**Authors:** Antonella Diamanti, Giacomo Calvitti, Diego Martinelli, Emma Santariga, Teresa Capriati, Giulia Bolasco, Lorenzo Iughetti, Arturo Pujia, Daniela Knafelz, Giuseppe Maggiore

**Affiliations:** 1 Hepatology Gastroenterology and Nutrition Unit, “Bambino Gesù” Children Hospital, 00165 Rome, Italy; teresa.capriati@opbg.net (T.C.); giulia.bolasco@gmail.com (G.B.); daniela.knafelz@opbg.net (D.K.); giuseppe.maggiore@opbg.net (G.M.); 2 Pediatric Unit, Department of Medical and Surgical Sciences for Mothers, Children and Adults, University of Modena and Reggio Emilia, 41121 Modena, Italy; giacomo.calvitti@gmail.com (G.C.); lorenzo.iughetti@unimore.it (L.I.); 3 Metabolic Diseases Unit, “Bambino Gesù” Children Hospital, 00165 Rome, Italy; diego.martinelli@opbg.net; 4 Clinical Nutrition Unit, Department of Medical and Surgical Sciences, University Magna Graecia, 88100 Catanzaro, Italy; emma_s@hotmail.it (E.S.); pujia@unicz.it (A.P.); 5 Medical Sciences Department Ferrara University, 44121 Ferrara, Italy

**Keywords:** intestinal failure, enteral nutrition, parenteral nutrition

## Abstract

Background: Intestinal failure (IF) is defined as reduction in functioning gut mass below the minimal amount necessary for adequate digestion and absorption. In most cases, IF results from intrinsic diseases of the gastrointestinal tract (digestive IF) (DIF); few cases arise from digestive vascular components, gut annexed (liver and pancreas) and extra-digestive organs or from systemic diseases (non-digestive IF) (NDIF). The present review revised etiology and treatments of DIF and NDIF, with special focus on the pathophysiological mechanisms, whereby NDIF develops. Methods: We performed a comprehensive search of published literature from January 2010 to the present by selecting the following search strings: “intestinal failure” OR “home parenteral nutrition” OR “short bowel syndrome” OR “chronic pseudo-obstruction” OR “chronic intestinal pseudo-obstruction” OR “autoimmune enteropathy” OR “long-term parenteral nutrition”. Results: We collected overall 1656 patients with well-documented etiology of IF: 1419 with DIF (86%) and 237 with NDIF (14%), 55% males and 45% females. Among DIF cases, 66% had SBS and among NDIF cases 90% had malabsorption/maldigestion. Conclusions: The improved availability of diagnostic and therapeutic tools has increased prevalence and life expectancy of rare and severe diseases responsible for IF. The present review greatly expands the spectrum of knowledge on the pathophysiological mechanisms through which the diseases not strictly affecting the intestine can cause IF. In view of the rarity of the majority of pediatric IF diseases, the development of IF Registries is strongly required; in fact, through information flow within the network, the Registries could improve IF knowledge and management.

## 1. Introduction

The term “intestinal failure” (IF) was defined originally by Fleming and Remington in 1981 to describe a state of “reduction in functioning gut mass below the minimal amount necessary for adequate digestion and absorption of food” [1]. Therefore, according to IF guidelines in adults, a “decreased absorption of macronutrients and/or water and electrolytes due to loss of gut function and need for Parenteral Nutrition (PN) should be both simultaneously present to define IF [2]. In children, IF derives from several diseases needing PN guaranteeing at least 75% of caloric requirements for not less than 1 month or at least 50% for not less than 3 months [3].

In most cases, IF is caused by diseases intrinsic to the gastrointestinal tract (digestive IF); few cases arise from digestive vascular components, gut annexed (liver and pancreas) and extra-digestive organs or from systemic diseases (non-digestive IF) [3,4,5,6,7,8,9,10,11,12,13,14,15,16,17,18,19,20,21,22,23,24]. The distinction between digestive IF (DIF) and non-digestive IF (NDIF) was first reported in a survey on Home Parenteral Nutrition (HPN) by the French group in 2007 [4] and confirmed in a later survey by the same group in 2016 [25].

Despite the multiplicity of diseases recognized as causes of IF, the pathophysiological mechanisms through which they trigger the gastrointestinal dysfunction are few and are common to DIF and NDIF. Three main pathophysiological mechanisms are recognized in children: short bowel syndrome (SBS), dysmotility and malabsorption/maldigestion. SBS derives from reduced intestinal length following neonatal or post-neonatal resections due to congenital or acquired gut diseases [26]. Dysmotility includes each disorder of muscular layers or enteric nervous system impeding the physiologic flux of intestinal content [15]. Finally, malabsorption/maldigestion derives from bowel pathologies not related with reduced length or impaired enteric muscular or nervous system [4,5,6,7,8,9,10,11,12,13,14,16,18,19,20,21,23]. While it is clear how DIF can develop from diseases directly affecting the intestine, it is instead less obvious and recognized how NDIF could develop. Furthermore, NDIF is generally the consequence of rare diseases beginning early in childhood and poorly understood. Therefore, we planned the present review to revise etiology and treatments of DIF and NDIF, with special focus on the pathophysiological mechanisms whereby NDIF develops.

## 2. Materials and Methods

### 2.1. Search Strategy

We performed a comprehensive search of published literature from January 2010 to the present on the PubMed database by selecting the following search strings: “intestinal failure” OR “home parenteral nutrition” OR “short bowel syndrome” OR “chronic pseudo-obstruction” OR “chronic intestinal pseudo-obstruction” OR “autoimmune enteropathy” OR “long-term parenteral nutrition”. References retrieved from pertinent articles were also included.

Results were filtered according to:

(a) Age: 0–18 years; (b) Species: Humans; (c) Language: English.

### 2.2. Inclusion Criteria

(1) SBS, dysmotility and malabsorption/maldigestion deriving from disorders of digestive vascular components, gut annexed organs (liver and pancreas) and extra-digestive organs or from systemic diseases and,

(2) Documented PN treatments.

### 2.3. Exclusion Criteria

(1) Unclear primary diagnosis;

(2) PN started as exclusive nutritional support without mention of underlying gastrointestinal dysfunction;

(3) Duplicate articles from the same center.

Three authors (AD, GC, ES) separately screened the studies for eligibility. Articles were screened in two stages. First, titles and abstracts were reviewed to identify potentially relevant articles. Full texts of those abstracts which met the inclusion criteria were retrieved and independently reviewed in the second stage of the assessment.

### 2.4. Data Extraction, Synthesis and Analysis

Data obtained from the selected articles were gathered and entered into tables. The following information was collected: author; year; publication; Country; patients’ number; gender; primary diseases for each IF case.

### 2.5. Endpoints

(1) Primary endpoints were etiology of DIF and NDIF and pathophysiological mechanisms whereby NDIF develops;

(2) Secondary endpoint was the therapeutic strategy of IF.

Etiology of DIF and NDIF was based on the systematic literature search; pathophysiological mechanisms and treatment strategies were drawn from articles included in the systematic review as well as from further pertinent and relevant papers.

## 3. Results

**Figure 1:**
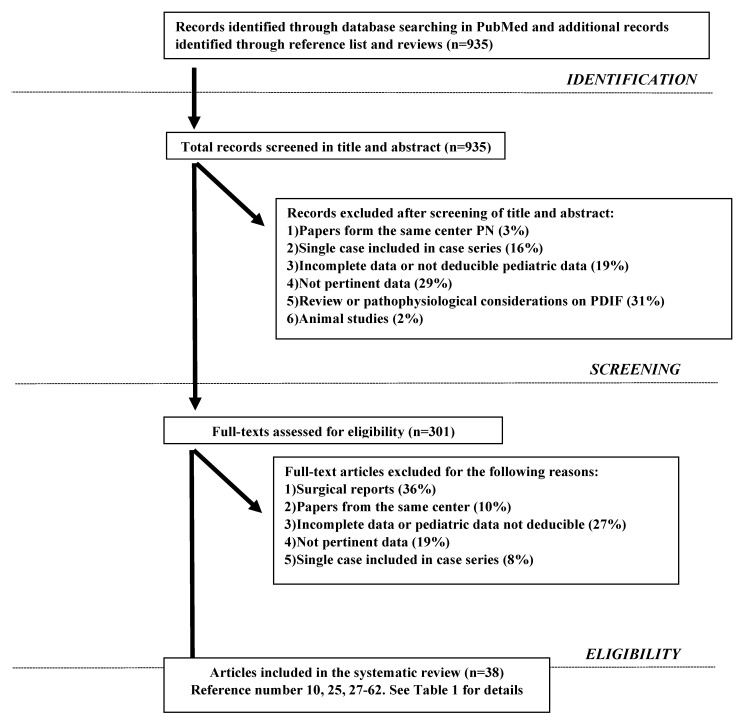
Search Strategy.

The systematic literature search identified 935 potentially relevant articles that matched the search criteria (see Figure 1). After considering our inclusion and exclusion criteria 38 articles were selected (See Figure 1 and Table 1) and 2052 patients were collected. However, all cases where etiology was unclear and where PN was used as nutritional support, not clearly needed for gastro-intestinal dysfunction, were excluded. In detail we collected overall 1656 patients with well-documented etiology of IF: 1419 with DIF (86%) and 237 with NDIF (14%), 55% males and 45% females. Etiology and pathophysiological mechanisms in DIF and NDIF are reported in Table 2 and Table 3, respectively; as shown, 66% of all cases of DIF were due to SBS while 90% of all cases of NDIF derived from malabsorption/maldigestion.

### 3.1. Pathophysiological Mechanisms of NDIF

#### 3.1.1. Intestinal Fistulas

Interestingly, our review identified only one patient with high-output fistula as cause of NDIF. The patient was a 15-year-old boy with Ehlers-Danlos syndrome with history of chronic constipation who developed megacolon, intestinal perforation and post-operative multiple entero-cutaneous fistulas. The high output from fistula required long-term PN [30]. This is the first case of IF due to fistula in children; however, it could be argued that the true cause of IF was the severe dysmotility complicated by intestinal perforation and entero-cutaneous fistula [30].

#### 3.1.2. Short Bowel Syndrome (SBS)

*Mesenteric ischemia*. Interrupted blood flow in a bowel area supplied by superior or inferior mesenteric artery can result in intestinal infarction and necrosis needing resection, often causing SBS. Mesenteric ischemia can derive from thromboembolic occlusion or vasospasm and it can be confused with necrotizing enterocolitis. Cardiac and abdominal surgery are the main triggers for non-occlusive mesenteric ischemia [63,64,65,66,67]. Kawasaki disease [50] has also been reported as cause of mesenteric occlusive ischemia in a 7-month-old infant. This infant developed digestive symptoms (diarrhea and vomiting) up to clear signs and symptoms of abdominal obstruction combined with bilious vomiting and imaging of small-bowel occlusion with superior mesenteric and splenic ischemia. He underwent intestinal resection with reduced bowel length which caused SBS with long-term dependence on PN.

*Meconium ileus*. It is the earliest clinical manifestation of cystic fibrosis, which presents as neonatal bowel obstruction of the distal small bowel in a subset of infants with severe cystic fibrosis transmembrane conductance regulatory mutations and pancreatic insufficiency [68]. In the simple form, viscid meconium physically obstructs terminal ileum that induces small intestine obstruction and dilation [68]. In the complex form the dilation is complicated by prenatal volvulus, ischemic necrosis, intestinal atresia, or perforation and extrusion of the meconium into the peritoneum [68]. Cystic fibrosis transmembrane conductance regulatory is responsible for both Cl−and HCO3–excretion; HCO3–plays a relevant role in chelating Ca2+ associated with the tight matrix of normally exocytosed mucins within the gut lumen, contributing to form normal and well-hydrated mucus [69]. Abnormal cystic fibrosis transmembrane conductance regulatory results in abnormal HCO3 secretion and consequent decreased luminal pH. This creates an acidic and dehydrated environment in which the tight matrix of exocytosed mucins is not disrupted appropriately, resulting in thick and dehydrated mucus [69]. The abnormally acidic luminal environment also increases stool albumin, minerals and protein-bound carbohydrates [68,69,70].

*Multiple atresia*. Multiple intestinal atresia, early bowel inflammation and severe combined immunodeficiency have been found associated to tetratricopeptide repeat domain 7A mutations [32,54,71,72,73,74,75]. Proteins encoded by tetratricopeptide repeat domain 7A are involved in polarization and differentiation of intestinal and likely thymic epithelial cells; mutations in this domain dysregulate the distribution of α-integrin and actin in the epithelial surface, leading to tissue architecture disorganization from fetal stage [71]. Multiple intestinal atresia and stenosis requires early surgery and intestinal resection causing SBS [54,71,73,74].

#### 3.1.3. Dysmotility

*Esophageal dysmotility, gastric outlet obstruction, small-bowel obstruction.* This pattern of dysmotility can occur in patients with eosinophilic gastroenteritis, a digestive disorder characterized by gastric and intestinal eosinophilic infiltration. Clinical phenotypes can differ according to gut layers at highest eosinophilic infiltration [76]. Muscle layer infiltration, ranging from 13 to 70% of all cases, affects stomach and duodenum and it can result in gastrointestinal occlusion and short term IF [76]. Chronic granulomatous disease involves digestive tracts in up to 50% of all cases leading, in some cases, to esophageal dysmotility, gastric outlet obstruction and small-bowel obstruction requiring short term PN [77].

*Alternating diarrhea and constipation*. Neonatal onset of alternating diarrhea and constipation episodes can cause IF as recognized in MEDNIK (mental retardation, enteropathy, deafness, neuropathy, ichthyosis and keratodermia) syndrome [78]. MEDNIK syndrome combines clinical and biochemical signs of the two classic disorders of copper metabolism: Menkes’s disease and Wilson’s disease [78,79].

*Recurrent paralytic ileus.* Gitelman syndrome, an autosomal recessive kidney tubule disorder with chronic hypokalemia, metabolic alkalosis, hypocalciuria and hypomagnesemia, can be cause of recurrent paralytic ileus [80]. In the present review two patients had recurrent paralytic ileus due to Gitelman’s syndrome [29]. Interestingly the present review identified a single case of electrolytes imbalance resulting in recurrent paralytic ileus following chronic diuretics administration in the context of pediatric falsification in Munchausen by proxy [29]. The need for increasingly invasive means of nutritional support due to intestinal dysfunction has indeed been reported as a potential feature of pediatric falsification [81].

*Severe MRGE delayed gastric emptying and jejunal dysmotility.* Amyoplasia, the most common form of arthrogryposis due to fatty–fibrous replacement of muscle [82], can be complicated by severe neuro-enteric dysfunction, which needs increasingly invasive means of nutritional support up to PN. [29]. Sanjad-Sakati syndrome is instead an autosomal recessive disorder which causes hypoparathyroidism, recurrent hypocalcemia, recurrent paralytic ileus, congenital growth retardation, seizures and typical facial dysmorphism [29,61]. It has been found associated to early development of IF requiring PN in one young girl [29].

*Pediatric intestinal pseudo-obstruction (PIPO).* PIPO has been reported associated to the Sanjad-Sakati syndrome [61] (see above) as well as to the Treacher Collins syndrome, which is caused by mutations in genes involved in neuroepithelial apoptosis during embryogenesis [38]. The child with Treacher Collins syndrome has been reported in the present review to have nutritional difficulties and digestive intolerance since birth; PIPO was suspected during childhood based on jejunal feeding intolerance and need for total PN. Histopathological confirmation was achieved on surgical rectal findings that showed enlarged ganglionic myenteric plexus [38]. The second patient with PIPO affected by Sanjad-Sakati syndrome was firstly evaluated for intermittent abdominal distension, bilious vomiting, and constipation when he was 6 years old. Plain abdominal radiographs and contrast barium swallow with follow-through showed dilated loops of intestine. Then, he underwent laparotomy due to increasing abdominal distension and respiratory difficulty, that excluded any identifiable structural causes of obstruction [61]. Histopathology found fibrotic changes of the longitudinal smooth muscle layer consistent with visceral myopathy. The child had satisfactory weight gain and maintained metabolic balance with total PN [61].

#### 3.1.4. Malabsorption/Maldigestion

*Protein losing enteropathy (PLE).* PLE is the most common pathophysiological mechanism leading to IF for malabsorption/maldigestion. PLE can occur in the context of primary or secondary disorders of lymphatics, which causes leakage of protein-rich chyle into the intestinal lumen, enteric protein loss, hypoalbuminemia, hypoproteinemia, lymphopenia, low fat-soluble vitamins deficiency and increased concentration of fecal α1-antitrypsin [19,20,21]. Primary intestinal lymphangiectasia is the congenital dilation of intestinal lymphatics [27,83,84]. The first case was reported by Waldmann et al. [27,85]; since then, nearly 200 cases of primary intestinal lymphangiectasia have been globally reported [27,83,86]. The classical symptoms are bilateral or unilateral lower limb edema, intermittent diarrhea, steatorrhea and fat-soluble vitamin deficiency, but pleural effusion or ascites can also develop [86]. Intestinal lymphangiectasia in Hennekam syndrome can also include genitalia and face edema, facial dysmorphisms and mental retardation [87]. Fontan pathway obstruction, pulmonary artery branch stenosis, increased pulmonary vascular resistance, elevated atrial pressures related to atrio-ventricular-valve regurgitation, arrhythmias and diastolic dysfunction can cause lymphatics dilation due to high systemic venous pressure, generally in infants younger than 3 years old [19,21,88,89]. Interestingly, following Fontan operation leakage of liver lymph through dilated hepato-duodenal lymphatic connections in duodenum has been also demonstrated [90]. Graft Versus Host Disease (GVHD) can develop as complication of allogenic hematopoietic stem cell transplantation (HSCT) [91,92] and is a further cause of PLE. Acute GVHD most commonly involves skin, liver and gastrointestinal tract in up to 60% of patients [93,94,95]. Gastrointestinal involvement may result in PLE and persistent anorexia, secretory diarrhea, abdominal pain and/or hemorrhage [96,97]. Furthermore, the subtype of eosinophilic gastroenteritis, involving only the mucosa layer of the gut can lead to PLE [76]. Finally, congenital disorders of glycosylation, a heterogeneous group of rare genetic disorders due to defects in protein, lipid or proteoglycan glycosylation, may present as severe PLE [98,99,100,101,102,103,104,105].

*Autoimmune enteropathy*. Autoimmune enteropathy has now been recognized as part of more complex pictures of immunodeficiency, such as immune dysregulation, polyendocrinopathy, enteropathy, X-linked syndrome (IPEX) [49] and therefore, it may be regarded as cause of NDIF [24]. In the past autoimmune enteropathy has been considered instead a form of DIF; in such perspective Unsworth and Walker-Smith first described a sub-group of infants with severe and protracted diarrhea, not responding to dietary restriction, with circulating gut autoantibodies and/or associated autoimmune diseases and lack of severe immunodeficiency [106]. In the present review two patients with autoimmune enteropathy were affected by IPEX, a primary immunodeficiency caused by mutations in FOXP3 gene, which encodes an essential transcription factor required for maintenance of thymus-derived regulatory T cells [107]. IPEX occurs in infancy with type-1 diabetes mellitus, autoimmune thyroiditis, autoimmune hemolytic anemia and a variety of skin lesions, including eczema, ichthyosiform dermatitis, psoriatic dermatitis and alopecia universalis [107]. Protracted high-volume diarrhea, due to autoimmune enteropathy, is generally the major presenting feature [107].

*Inflammation*. Primary immunodeficiency can lead to IF by severe inflammation. In particular, tetratricopeptide repeat domain 7A mutations [32,54] (see also above) can cause severe exfoliate apoptotic enterocolitis and perianal fistula responding to steroids [32]. Furthermore, chronic inflammation is often seen in patients with chronic granulomatous disease (see above) and it is related to exuberant and persistent tissue granuloma formation [108,109,110], clinically and radiographically indistinguishable from Crohn’s disease [109,111,112,113,114]. Cystic fibrosis patients can also develop non-specific intestinal inflammation. The non-functional cystic fibrosis transmembrane conductance regulatory on the apical membrane of secretory and absorptive epithelial cells decreases chloride and water secretion resulting in precipitation of secretions, intra-ductal obstructions, inflammation, tissue damage, and fibrosis [115]. Capsule endoscopy can show mucosal ulceration and erythema [116,117].

*Intractable osmotic diarrhea*. Osmotic intractable diarrhea (stool anion gap > 50, fecal pH < 6 and fasting test positive) [118] can occur in enteric anendocrinosis and enteric dysendocrinosis. Enteric anendocrinosis is due to mutations in Neurogenin-3, a basic helix-loop-helix transcriptional factor that drives the development of endocrine cell in both the pancreas and intestine [119,120,121]. These mutations cause lacking intestinal entero-endocrine cells [39] and early or later presentation of diabetes mellitus [119,120,121]. Enteric dysendocrinosis is caused by mutations of the gene encoding for prohormone convertase 1/3, a calcium-dependent serine endoprotease essential for the conversion of prohormones in the bioactive form. It is expressed in endocrine cells in the gut, in arcuate and paraventricular nuclei of hypothalamus, and in β-cells of the pancreas, where it has a well-defined role in processing proinsulin. Neonates with nonfunctional prohormone convertase 1/3 show IF and additional endocrine abnormalities such as diabetes insipidus, growth hormone deficiency, primary hypogonadism, adrenal insufficiency, and hypothyroidism [53].

### 3.2. Management

The main thread of nutritional strategy in any case of IF is to integrate the maximum tolerated amount of Enteral Nutrition (EN) with the ongoing PN support. The objective of nutritional work-up should be reaching intestinal autonomy, which depends on the type of IF. From a prognostic point of view Shaffer et al. defined as Type I IF an acute, short-term, and usually self-limiting condition [3]. Type II is instead considered as prolonged acute condition, requiring complex multi-disciplinary care and PN over weeks or months. It is possible that some forms of Type II IF may evolve in Type III IF. Type III IF, also called chronic IF, occurs in metabolically stable patients, who require PN over months or years and may be reversible or irreversible [2].

The strategies to treat/improve/revert IF may be summarized as follows:

(1) Specific treatments that could reverse the underlying disease responsible for IF (e.g., hematopoietic stem cell transplantation in primary immunodeficiency);

(2) Treatments allowing to improve/revert the pathophysiological mechanism leading to IF (e.g., nutritional treatments, steroids and octreotide in PLE);

(3) Treatments for chronic DIF and NDIF able to reduce PN dependency over time (e.g., analog of glucagon like peptide 2 and surgical procedures to increase mucosal surface area in SBS or prokinetic drugs in PIPO [15,21,22,26,27,29,49,62,81,82,83,84,85,86,90,122,123,124]

The advances in EN tolerance over time and the progressive decreasing in calories and fluids provided by PN will be the clinical markers of successful application of specific and non-specific therapies.

In Table 4 we report the main therapeutic options available to combine with nutritional management in DIF and NDIF.

## 4. Discussion

The present review provides new insights on NDIF only occasionally and not systematically focused on literature. We observed that overall, 14% of patients require prolonged PN treatments due to diseases not strictly inherent to intestine. In 2007 Colomb and coll [3] found a prevalence of NDIF of 24%; this survey included, nevertheless, patients without clear intestinal dysfunction but requiring PN as nutritional support as well as patients with metabolic diseases, probably including mitochondrial disorders, now considered as causes of DIF [15]. Mitochondrial disorders are multi-systemic diseases affecting predominantly organs or systems with high-energy metabolism such as central nervous system, heart and skeletal muscle [125]. Gastrointestinal complaints could be gastrointestinal dysmotility, gastroparesis, progressive intestinal pseudo-obstruction, abdominal pain, dilation and dysmotility of the oesophagus, stomach and the small intestines, and malabsorption with progressive malnutrition [126]. Mitochondrial neurogastrointestinal encephalomyopathy, the prototype of mitochondrial IF with pseudo-obstruction, is a rare autosomal recessive disease due to defects in the thymidine phosphorylase gene, encoding the enzyme responsible for the conversion of deoxynucleosides (deoxythymidine and deoxyuridine) [127]. Pathological accumulation of deoxythimidine and deoxyuridine leads to the typical manifestations of the disease [127,128]. Digestive symptoms are early satiety, nausea, vomiting, dysphagia, gastro-esophageal reflux, abdominal pain and pseudo-obstruction or diarrhea, probably due to dysfunctional network of intestinal Cajal cells, that is the pacemakers of the gut [126].

A more recent survey by the French Group [25] found a prevalence of NDIF of 14%. Therefore, from an epidemiological perspective we can conclude that the prevalence of NDIF may be established as for more than 10%.

In more general terms the prevalence of pediatric IF has dramatically increased over time. Previous papers reported IF prevalence ranging from 2 and 6.8 per 1,000,000 of inhabitants in developed Countries [13,128,129]. In Italy in 2016, we observed prevalence and incidence of 14.12 and 1.41 par million inhabitants ≤19 years, respectively [36]. A comparable trend has been demonstrated in UK where IF prevalence has risen from 4.4/million in 1993 to 13.9/million in 2010 and to 14.5/million in 2012 [130].

Management of IF requires a close interplay between several actors in a multidisciplinary scenario where technical skills should be shared to offer the best and most tailored treatment to each patient. Nutritional work-up is only a part of the complex management of IF patients and it does not differ between DIF and NDIF. IF patients have highly specialized needs, and their care should be provided by centers of excellence providing sufficient surgical, medical, dietetic and nursing expertise to treat long-term IF and home parenteral nutrition (HPN) [36]. HPN should be proposed as soon as possible to the family when indicated [36]. IF patients who have developed severe complications that make PN unsafe should be cared in centers with expertise in IF and intestinal transplantation [131,132].

Over the last 25 years, the outcome of IF has transformed from almost certain death in childhood to a high chance of survival into adult life, even when still PN-dependent [133]. Therefore, the challenge is now to find therapeutic approaches able to reduce the dependency on PN over time. Relevant advances in the most recent years, have been seen in the field of immunodeficiencies and SBS, together accounting for the 63.5% of all causes of IF in the present review.

It is now known that many forms of autoimmune enteropathy, in the past considered as causes of DIF, are clinical manifestations of primary immunodeficiency [24]. The main curative treatment of IF combined with immunodeficiency is the hematopoietic stem cells transplantation [49]; therefore, the prognosis of autoimmune enteropathies has strongly changed, because PN is now considered as bridge to hematopoietic stem cells transplantation and not as a long-term treatment as in the past [24].

Furthermore, surgery and hormonal therapy have enriched the therapeutic armamentarium of SBS.

The impact of surgery on weaning off PN was assessed by a recent systematic literature review [122]. It found that surgery has low benefit in terms of intestinal adaptation and therefore it should not be proposed to all patients with SBS, but only to selected candidates. Main requirements to refer for surgery should be radiologically evident bowel dilation associated with signs or symptoms of small bowel bacterial overgrowth, such as failure of advancing enteral nutrition and poor growth. Early signs of intestinal failure-associated liver disease (IFALD) should also be considered, if associated with intestinal dilation, as a factor in favor of eligibility for surgery [122].

Clinical trials have proven safety, tolerability, and efficacy of the recombinant form of GLP-2 in the treatment of SBS-intestinal failure in children [123,134]. In the first published case series, outside clinical trials, Ramos Boluda et al. [135] reported a substantial improvement in the outcome for SBS children and they considered their outcome even better than that reported in the paediatric clinical pivotal study. Therefore, in the future, surgery and hormonal therapy, tailored on the single patient, could be promising strategies to improve the prognosis of SBS.

## 5. Conclusions

The improved availability of diagnostic and therapeutic tools has increased both the prevalence and life expectancy of rare and severe diseases responsible for IF. Current knowledge about pathophysiological mechanisms of the diseases has led to identify previously unrecognized extra-digestive causes of IF. The present review greatly expands the spectrum of knowledge on the pathophysiological mechanisms through which the diseases not strictly affecting the intestine can cause IF. In view of the rarity of the majority of pediatric IF diseases, the development of IF Registries is strongly required; in fact, through information flow within the network the Registries could not only improve the knowledge about the causes of IF, but also allow management to be shared.

## Figures and Tables

**Table 1:** Summary of the studies selected for the review.

REF.	Author (year)	Country	Study-Period	*n*° of Patients	NDIF (%)	DIF (%)
[27]	Prasad D (2019)	India	2017–2017	6	6 (100)	0
[28]	LaRusso K (2019)	Canada	2006–2018	37	1 (3)	36 (97)
[29]	Diamanti A (2019)	Italy	1988–2018	48	9 (19)	39 (81)
[30]	Zemrani B (2019)	Australia	2018	1	1 (100)	/
[31]	Zapata Olivares Y (2019)	Chile	2016–2017	46	3 (7)	43 (93)
[32]	Fayard J (2018)	France	2000–2017	65	65 (100)	/
[33]	Raphael BP. (2018)	USA	2001–2016	78	2 (3)	76 (97)
[34]	Gunnar R. (2018)	Finland	2012–2015	49	/	49 (100)
[35]	Merras-Salmio L. (2018)	Finland	1984–2017	100	3 (3)	97 (97)
[36]	Diamanti A. (2017)	Italy	2016	145	23 (16)	122 (84)
[37]	Blotte C. (2017)	USA	2012–2016	36	/	36 (100)
[38]	Giabicani E. (2017)	France	2016	1	1 (100)	/
[39]	German-Diaz M. (2017)	Spain	2016	1	1 (100)	/
[40]	Gonzalez-Hernandez J. (2017)	USA	1999–2012	71	/	71 (100)
[41]	Stýblová J. (2017)	Czech Republic	1995–2011	66	3 (5)	63 (95)
[42]	Hashimura Y. (2016)	Japan	2016	1	/	1 (100)
[25]	AbiNader E. (2016)	France	2000–2013	251	34 (14)	217 (86)
[40]	Gonzalez-Hernandez J. (2016)	USA	2010–2014	30	/	30 (100)
[43]	Furtado S. (2015)	CANADA	2007–2012	55	/	55 (100)
[44]	Pichler J. (2015)	UK	2002–2010	71	7 (10)	64 (90
[45]	Mirabel-Chambaud E (2015)	France	2007–2014	183	19 (10)	164 (90)
[46]	Mezoff EA (2015)	USA	2010–2013	30	3 (10)	27 (90)
[47]	Neelis EG (2015)	Netherland	2013	37	/	37 (100)
[48]	Pichler J (2014)	UK	2006–2010	127	46 (36)	81 (64)
[49]	Singhi AD (2014)	USA	1996–2013	14	/	14 (100)
[50]	Godart F. (2014)	France	2014	1	1 (100)	/
[10]	Hizarcioglu-Gulsen H. (2014)	Turkey	2000–2010	60	37 (62)	23 (38)
[51]	Duro D. (2014)	US	2013	28	/	28 (100)
[52]	Courtney-Martin G. (2014)	Canada		27	3 (11)	24 (89)
[53]	Martin GM (2013)	USA	2004–2012	14	14 (100)	/
[54]	Samuels ME. (2013)	Canada	2013	5	5 (100)	/
[55]	Ubesie AC. (2013)	USA	2007–2012	123	/	123 (100)
[56]	Derepas C. (2013)	Canada	2012	13	2 (15)	11 (85)
[57]	Sadlier C. (2013)	UK	2007–2010	36	13 (36,1%)	23 (63.8%)
[58]	Pieroni KP. (2013)	USA	2007–2011	14	1 (7)	13 (93)
[59]	Javid PJ. (2011)	USA	2005–2009	62	/	62 (100)
[60]	Diamanti A. (2010)	Italy	2005–2007	24	1 (4)	23 (96)
[61]	Pal K. (2010)	Saudi Arabia	2009	1	1 (100)	/

**NDIF**: Non-digestive Intestinal Failure; **DIF**: Digestive Intestinal Failure.

**Table 2:** Summary of the causes of Digestive Intestinal Failure (DIF).

	Ref. *n*, Etiology	*n*° (%)
Digestive IF		1419
***Short Bowel Syndrome***	[25,28,31,32,33,34,35,36,37,40,41,43,44,45,46,47,48,51,52,55,56,57,58,59,60,62]	**943 (66)**
	*Necrotizing enterocolitis*	364
	*Volvulus*	201
	*Gastroschisis*	191
	*Atresias*	177
	*Double malformations*	4
	*Spontaneous perforation*	3
	*Omphalocele*	3
***Dismotility***	[10,25,28,29,31,33,34,35,36,40,41,44,45,46,47,48,52,55,56,57,58,59,60,61]	**285 (20)**
	*Chronic intestinal pseudo-obstruction*	154
	*Hirshprung disease*	107
	*Mitocondrial diseases*	21
	*Celiac disease*	2
	*Gastroschisis*	1
***Malabsorption/maldigestion***	[10,28,31,33,35,37,40,41,42,45,46,48,51,54,55,57,58,59]	**191 (14)**
	**Congenital enteropathy**	**93**
	*Not classified*	16
	*Tufting enteropathy*	41
	*Mycrovillous inclusion disease*	31
	*Trichoepatoentehric Syndrome*	5
	**IBD**	**49**
	**Autoimmune enteropathy**	**30**
	**Selective malabsorption**	**19**
	*Glucose-galactoses*	*12*
	*Sucrase-isomaltases*	*1*
	*Diacylglycerol acyltransferase (DGAT 1) deficiency*	*2*
	*Abetalipoproteinemia*	*4*

**IF:** Intestinal Failure, **IBD:** Inflammatory Bowel Diseases.

**Table 3:** Summary of the causes of Non-digestive Intestinal Failure (NDIF).

	Ref. *n*, Etiology	*n* (%)
Non-Digestive IF		237
***Intestinal Fistulas***	[30]	**1 (0.4)**
	**Ehlers-Danlos Syndrome**	**1**
***SBS***	[25,36,41,46,50,57]	**9 (3,8)**
	**Trauma**	2
	**Cardiovascular diseases**	5
	*Mesenteric ischemia*	*4*
	*Kawasaky Syndrome*	*1*
	**Pancreatic diseases**	2
	*Meconium ileus (Cystic Fibrois)*	*2*
***Dismotility***	[29,36,38,52,57,61]	**14 (5.9)**
	**Neurodisabling diseases**	**8**
	*Neuroevolutive disorders*	*7*
	*Teacher Collins Syndrome*	*1*
	**Chronic electrolytes/minerals imbalances**	**4**
	*Gitelman Syndrome*	*2*
	*Sanjad-Sakati Syndrome*	*2*
	**Fabricated disease**	**1**
	**Conditions affecting GI smooth muscle**	**1**
	*Congenital amioplasia*	*1*
***Malabsorption/maldigestion***	[10,25,27,28,31,32,33,35,36,39,41,42,43,44,45,46,48,49,51,52,53,54,57]	**213 (89.9)**
	**Primary immunodeficiency**	**109**
	*Not classified*	*39*
	*IPEX Syndrome.*	*2*
	*Chronic granulomatosis*	*1*
	*Di George Syndrome*	*1*
	*TTC7A mutations*	*66*
	**Cancer + HSCT**	**37**
	*Not classified*	35
	*GVHD*	2
	**Pancreatic diseases (Pseudocystis and CF)**	**9**
	**Metabolic diseases**	**2**
	*Protein Glycosilation deficiency*	*1*
	*Mednik Syndrome*	*1*
	**Entero-endocrine**	**17**
	*Enteric anendocrinosis (Mut. in Neurogenin 3 gene)*	*2*
	*Pro-protein convertase 1/3 (PC1/3) deficiency*	*15*
	**Allergic enteropthy**	**13**
	**Cardiovascular diseases**	**26**
	*Congenital hearth defects*	*2*
	*Intestinal lymphangectasia*	*24*

**IF**: Intestinal Failure; **GI**: gastrointestinal; **IPEX**: immune dysregulation, polyendocrinopathy, enteropathy, X-linked; **TTC7A**: tetratricopeptide repeat domain 7A **HSCT:** hematopoietic stem cells transplantation; **GVHD**: Graft versus host disease.

**Table 4:** Therapeutic approach in Intestinal Failure.

SPECIFIC TREATMENTS FOR PRIMARY DISEASES	
***Primary disease***	**Treatments**
**Immunodeficiency**	HSCT
**Cystic Fibrosis**	CFTR modulators to correct the basic defect
**GVHD**	**Steroids is the first choice**; for patients who develop steroid refractory GVHD the following rescue therapies are available: pentostatin, antithymocyte globulin, alemtuzumab, infliximab, basiliximab/infliximab combination therapy, ruxolitinib, tocilizumab, brentuximab vedotin, vedolizumab
**Eosinophilic gastroenteritis**	(a) **Dietetic**: exclusion of the offending antigen (b) **Pharmacologic**: prednisone; budesonide; montelukast sodium, sodium cromoglycate, Ketotifen azathioprine and 6-mercaptopurine. Proton-pump inhibitors. Biologic drugs. Intravenous immunoglobulin. Interferon α. Microbiota transplantation (c) **Surgery** in presence of complications such as perforation, intussusception, or intestinal occlusion or when a full-thickness intestinal biopsy should be performed to establish the diagnosis
**NON-SPECIFIC TREATMENTS FOR SYMPTOMS OF IF**	
***Pathophysiological mechanism***	**Treatments**
**PLE**	***Standard treatments***: Low-fat diet (<25% of the caloric intake from fat), MCT supplementation, high protein diet (>2 gr/Kg/die), human albumin transfusion, vitamins, electrolyte supplements, gamma globulin infusions (if recurrent infections, preceded by low serum IgG), steroids and octreotide. ***Specific treatment for heart disorders at increased central venous pressure***: (a) **Pharmacologic**: heparin, budesonide, spironolactone, dopamine; (b) **Trans-catheter therapy**: Dilation and/or stenting of obstructions to systemic arterial, and systemic or pulmonary venous flow, embolisation of significant aorto-pulmonary collaterals, liver lymphatic embolization; (c) **Surgery**: Fontan revision, cardiac transplantation. ***Specific treatments for primary lymphangectasia***: tranexemic acid; anti-plasmin; propanololo; everolimus; eculizumab (in CD55 deficiency); surgical resection of segmental or localized disease.
**Inflammation and recurrent infections in immunodeficiency**	Steroids; immunosuppression; antibiotic and antifungal prophylaxis; prophylactic use of IFN-γ, infliximab in patients with colitis; immunoglobulin substitution.
**Inflammation in Cystic Fibrosis**	Careful management of enzyme usage, dysmotility and bacterial overgrowth
**NON-SPECIFIC TREATMENTS FOR CHRONIC IF**	
***Cause of chronic IF***	**Treatments**
**SBS**	(a) **Pharmacologic standard therapy**: anti-diarrheal agents, acid suppression medications, bile acid binding salts, and enteral antibiotics; (b) **Non-standard pharmacologic therapy**: teduglutide; (c) **Surgery** (longitudinal intestinal lengthening and tailoring and serial transverse enteroplasty procedures).
**PIPO**	(a) **Pharmacologic**: prokinetic medications at the discretion of the specialist caring for the patient; (b) **Surgery**intestinal resection, ostomy formation, ostomy revision, need for full thickness.

**IF**: Intestinal Failure; **HSCT**: hematopoietic stem cell transplantation; **CFTR**: cystic fibrosis transmembrane conductance regulatory; **GVHD**: graft versus host disease; **PLE**: protein losing enteropathy; **SBS**: short bowel syndrome; **PIPO**: pediatric intestinal pseudo-obstruction. See References [15,21,22,26,27,29,49,62,81,82,83,84,85,86,90,122,123,124].
